# *ADAMTS* proteoglycanases downregulation with impaired oocyte quality in PCOS

**DOI:** 10.20945/2359-3997000000321

**Published:** 2021-01-14

**Authors:** Sepide Gohari taban, Iraj Amiri, Massoud Saidijam, Sara Soleimani asl, Mahnaz Yavangi, Elham Khanlarzadeh, Nooshin Mohammadpour, Tayebe Artimani

**Affiliations:** 1 Hamadan University of Medical Sciences Anatomy Department School of Medicine Hamadan Iran Anatomy Department, School of Medicine, Hamadan University of Medical Sciences, Hamadan, Iran; 2 Hamadan University of Medical Sciences Endometrium and Endometriosis Research Center Hamadan Iran Endometrium and Endometriosis Research Center, Hamadan University of Medical Sciences, Hamadan, Iran; 3 Hamadan University of Medical Sciences Research Center for Molecular Medicine Hamadan Iran Research Center for Molecular Medicine, Hamadan University of Medical Sciences, Hamadan, Iran; 4 School of Medicine Department of Community Medicine Hamadan Iran Department of Community Medicine, School of Medicine, Hamadan, Iran

**Keywords:** *ADAMTS-4*, *ADAMTS-5*, PCOS, oocyte quality, progesterone receptors

## Abstract

**Objective:**

A disintegrin and metalloproteinase with thrombospondin motifs 4 (*ADAMTS-4*) and *ADAMTS-5* normal expression levels are essential for ovulation and subsequent fertilization. The objective of the present study was to assess expression pattern of these genes in cumulus cells (CCs) taken from patients with polycystic ovary syndrome (PCOS) and to investigate any possible relationship with the oocyte quality.

**Subjects and methods:**

*ADAMTS-4* and *-5* expression levels within CCs containing oocytes at the metaphase II (MII) and germinal vesicle (GV) stages, taken from 35 patients with PCOS and 35 women with normal ovarian function, were investigated using RT-qPCR. Moreover, possible correlations between *ADAMTS-4, ADAMTS-5*, and progesterone receptors (PRs) expression as well as oocyte quality were evaluated.

**Results:**

ADAMTS-4 and -5 expression levels were dramatically diminished in the CCs of the PCOS patients when compared to the controls. *ADAMTS-4* and *-5* expression levels were correlated with each other and with the oocyte quality. Furthermore, lower expression levels of *ADAMTS-4* and *-5* in the PCOS patients were strongly correlated with the diminished PRs expression levels.

**Conclusions:**

Downregulation of *ADAMTS-4* and *-5* in the human CCs of the PCOS patients correlated with the decline in the PRs expression, and impaired oocyte quality may cause lower oocyte recovery, maturation, and fertilization rate.

## INTRODUCTION

**P**olycystic ovary syndrome (PCOS) is a complex multifactorial endocrine abnormality common among women at reproductive age (1). Impaired ovarian steroidogenesis and folliculogenesis, neuroendocrine axis dysfunction, changes in metabolism, insulin secretion and sensitivity, adipose cell dysfunction, and altered inflammatory factors are involved in PCOS pathogenesis (2). PCOS abnormalities in the intra-follicular milieu negatively influence the oocyte gene expression and oocyte cytoplasmic or nuclear maturation (3).

During the ovulation process, levels of extracellular matrix (ECM) components such as versican, aggrecan, brevican, and hyaluronic acid increase in ovaries following (luteinizing hormone) LH surge (4). LH induces expression of pivotal genes involved in ovulation, such as the progesterone receptor (PR) (5). Previous study highlighted a significantly lower expression of *PRs* in granulosa cells (GCs) of PCOS compared to controls (6). A series of proteinases such as matrix metalloproteinases and a disintegrin and metalloproteinase with thrombospondin motifs (ADAMTS) proteinases cause destruction and remodeling of ECM compounds (7). Binding of progesterone to PR on GCs results in a remarkable enhancement of *ADAMTS-1* expression, which breaks the follicle wall (4).

ADAMTS-1, *-4*, and *-5* have a role in degradation of brevican and versican and other ECM structures (4). Versican contributes to remodeling and maintenance of the ECM structural and functional integrity and movement of cumulus cells (CCs) (4). Versican proteolysis in the peri-ovulatory period causes cumulus-oocyte complex (COC) expansion, which is essential for successful ovulation (4,8). Follicle-stimulating hormone (FSH) provokes follicle growth and survival, and also, stimulates ADAMTS -1, -4, and -16 expression, indicating a mechanistic link between these proteolytic enzymes and follicle growth (9). Although expression of *ADAMTS-4, -5, -9, -16,* and *-17* was reported in ovaries, only *ADAMTS-1* has been studied extensively (4). *Adamts-4* and *-5* expression has been observed previously in the GCs and CCs of rodents and monkeys during folliculogenesis (9). Karakose and cols. suggested that *ADAMTS-1, ADAMTS-5, ADAMTS-9* aggrecanases, and interleukin molecules may play a role in PCOS pathogenesis (10).

Therefore, we assumed that *ADAMTS-4* and -5 dysregulation might be associated with oocyte abnormalities in PCOS patients. The present study aimed to assess *ADAMTS-4* and *-5* expression levels in the CCs of patients with PCOS and women with normal ovarian function during an IVF procedure. Moreover, possible correlations were investigated between *ADAMTS-4* and *-5* expression with oocyte quality.

## SUBJECTS AND METHODS

This cross-sectional study was carried out on 70 infertile women (35 women with PCOS and 35 women with normal ovulatory function) aged 18-39 years (mean age = 29.4 ± 5.3) undergoing intra-cytoplasmic sperm injection (ICSI) during 2016-2017. The exclusion and inclusion criteria for the control and PCOS groups were defined, as reported in previous study (11). All procedures used in the present study including measurement of the basal serum levels of FSH and LH, ovarian stimulation and CCs collection, RNA isolation, cDNA synthesis, and quantitative real-time PCR as well as evaluation of oocyte quality parameters were similar to those reported in the previous work of the authors (11). Briefly, for ovarian stimulation, PCOS and control patients recruited to the study were treated with a GnRH agonist in the mid-luteal phase of the previous menstrual cycle. Recombinant FSH (Gonal-F, Merck Serono, Switzerland) was used for ovarian stimulation. Ultrasound was implemented every 1-3 days to assess follicle development. Daily FSH doses could be adjusted according to the ovarian response after first 3-5 days of treatment. Moreover, 5000-10,000 IU of human chorionic gonadotropin (hCG, Choriomon, IBSA, Lugano, Switzerland) was administered following detection of at least three dominant follicles (diameter ≥18-20 mm). Oocyte retrieval was performed 34-36 h after hCG administration with transvaginal ultrasound-guided needle puncture. COCs were isolated through ultrasound-guided vaginal puncture. CCs surrounding oocytes were removed with strippers following a short time exposure to the hyaluronidase (SAGE, Trumbull, CT) at 37 °C. The oocytes were categorized into mature oocytes with the first polar body (metaphase II, MII) and immature oocytes at the germinal vesicle (GV) stage in accordance to the nuclear status. To form CCGV and C CMII groups for qPCR analysis, purified CCs were separately pooled from one of the patients. CCs were centrifuged at 800×g for 8 min at room temperature, and resulting pellets were used for RNA purification. The oocytes were injected using the ICSI procedure, and fertilization was confirmed 16-18 h after insemination when two pronuclei were appeared.

Primer sequences used in real time PCR (RT-PCR) analysis, are listed in Table 1. This study was a part of a project approved by the Ethics Committee of the Hamadan University of Medical Sciences (IR.UMSHA.REC.1394.499) (11). The data were analyzed using SPSS software version 16. The results are presented as mean ± SE. Comparison between the groups with normal distribution was performed using Independent Sample T-Test, and Mann-Whitney *U* test was used for nonparametric analyses. The relationship between the parameters was determined using Spearman coefficients. P-values less than 0.05 were considered statistically significant.

**Table 1:** Characteristics of the primers

Gene	Forward primer	Reverse primer	Accession number	Product size bp
*ADAMTS-4*	TTGGGGAGACGCTGCTACTA	TGTAACACGCCTAACAGGGC	NM_001320336.1	199
*ADAMTS-5*	TCGGGAGGATTTATGTGGGC	TGGAATCGTCATGGGAGAGG	NM_007038.4	173
B-actin	AAGATCAAGATCATTGCT	TAACGCAACTAAGTCATA	NM_001101.4	177
PR total	CTCATCCATACTTATCCTTCAC	TCCTTGTCCACTTCACTT	NM_001202474	206
*PR-B*	GGTAAGCCTTGTTGTATT	GGGTTGTAGATTTCACTC	NM_000926.4	85

## RESULTS

### Study population

Clinical characteristics and IVF-ET outcomes of the PCOS patients and the women with normal ovarian function are shown in Table 2. No considerable difference was noticed between the PCOS and control groups regarding age, duration of infertility, BMI, total rFSH dose, and FSH level. However, in agreement with the results of previous investigations, basal LH concentrations were higher in the PCOS patients than in the controls (p = 0.02). As shown in Table 2, the number of follicles punctured and oocytes retrieved in the PCOS patients was considerably higher than those in the control group (p < 0.0001 and p = 0.009, respectively). However, no significant difference was observed between the groups in terms of MII oocytes. Moreover, the oocyte quality parameters, as depicted in Table 2, were considerably higher in women with normal ovarian function when compared to the PCOS patients. In more details, oocyte recovery rate (p = 0.05), oocyte maturation rate (p = 0.01), and fertilization rate (p = 0.05) were markedly lower in the PCOS patients compared to the controls.

**Table 2:** Clinical characteristics and IVF-ET outcomes in PCOS and control groups

Variable	Controls	PCOS	P-value
Age (year)	30.8 ± 0.93	29.06 ± 0.83	0.1
BMI (kg/m^2^)	25.5 ± 0.76	26.3 ± 0.78	0.4
Duration of infertility (year)	5.59 ± 0.97	6.46 ± 0.76	0.4
Basal FSH (IU/L)	5.8 ± 0.45	6.8 ± 0.4	0.07
Basal LH (IU/L)	4.6 ± 0.29	7.08 ± 1.05	0.02
Total rFSH dose (IU)	17297 ± 88.3	15208 ± 77.8	0.08
Number of follicles punctured	7.6 ± 0.8	14.8 ± 1.6	<0.0001
Number of oocytes retrieved	7.5 ± 0.7	11.13 ± 1.03	<0.009
Oocyte recovery rate (%)	1.05 ± 0.06	0.86 ± 0.06	0.05
Number of MII oocytes	6.3 ± 0.7	7.6 ± 0.7	0.2
Number of GV oocytes	0.88 ± 0.18	3.07 ± 0.51	<0.0001
Oocyte maturation rate (%)	0.8 ± 0.03	0.7 ± 0.03	0.01
Fertilization rate (%)	0.57 ± 0.04	0.46 ± 0.03	0.05

PCOS: polycystic ovary syndrome; BMI: body mass index; MII: metaphase II. Data were presented as mean ± SEM and compared by Independent-Samples T-test.

### ADAMTS-4 and -5 expression in PCOS patients and women with normal ovarian function

**Figure 1:**
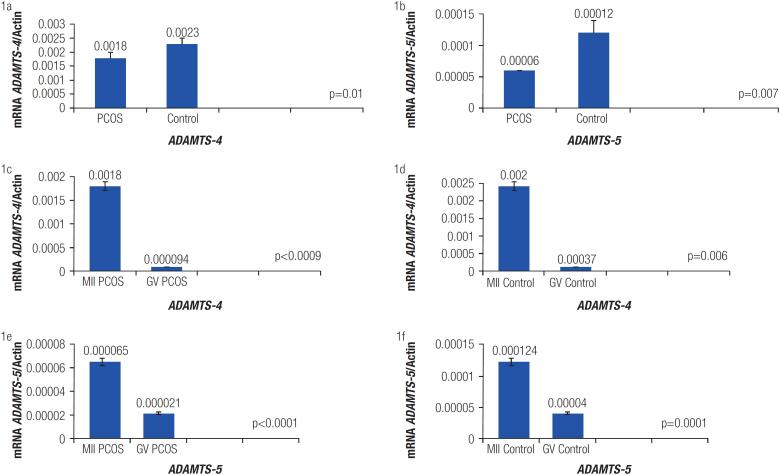
*ADAMTS-4* and *-5* expression in the cumulus cells of the polycystic ovary syndrome and control groups.

PCR analysis revealed significant expression of ADAMTS-4 and -5 genes in the human CCs. Quantitative real-time PCR demonstrated significantly higher *ADAMTS-4* mRNA expression levels in the control group compared to the PCOS women (p = 0.01, Figure 1a). Furthermore, *ADAMTS-5* mRNA expression levels were remarkably reduced in the PCOS patients when compared to women with normal ovarian function (p = 0.007, Figure 1b).

### Associations between *ADAMTS-4* and *-5* expression and oocyte maturation

To evaluate possible correlations between *ADAMTS-4* and *-5* expression with oocyte maturation, expression levels of these genes were assessed in CCs containing MII and GV oocytes. ADAMTS-4 mRNA expression levels were notably higher in CCs containing MII oocytes compared to CCs with GV oocytes in the both PCOS and control groups (p < 0.0009 and p = 0.006, respectively, Figures 1c and 1d). Moreover, *ADAMTS-5* expression levels considerably decreased in CCs with GV oocytes compared to CCs with MII oocytes in the both PCOS and control groups (p < 0.0001 and p = 0.001, respectively, Figures 1e and 1f).

### Associations between *ADAMTS-4* and *-5* expression and oocyte quality in PCOS patients

**Figure 2:**
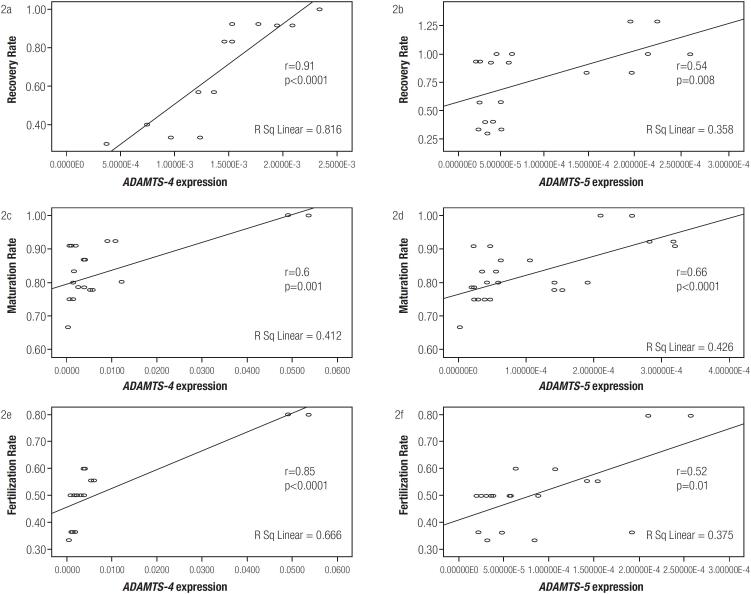
Correlations between ADAMTS-4 and -5 expression with recovery rate, maturation rate, and fertilization rate in the cumulus cells.

There were strong positive correlations between *ADAMTS-4* and -5 mRNA expression levels with the oocyte recovery rate (p < 0.0001, r = 0.91 and p = 0.008, r = 0.0.54, respectively, Figures 2a and 2b). In addition, *ADAMTS-4* and *-5* mRNA expression levels were markedly associated with the oocyte maturation rate (p = 0.001, r = 0.6 and p < 0.0.0001, r = 0.66, respectively, Figures 2c and 2d). Moreover, the fertilization rate was strongly correlated with *ADAMTS-4* and *-5* mRNA expression levels (p < 0.0001, r = 0.85 and p = 0.01, r = 0.52, respectively, Figures 2e and 2f).

### The association between ADAMTS proteoglycanases and PRs

**Figure 3:**
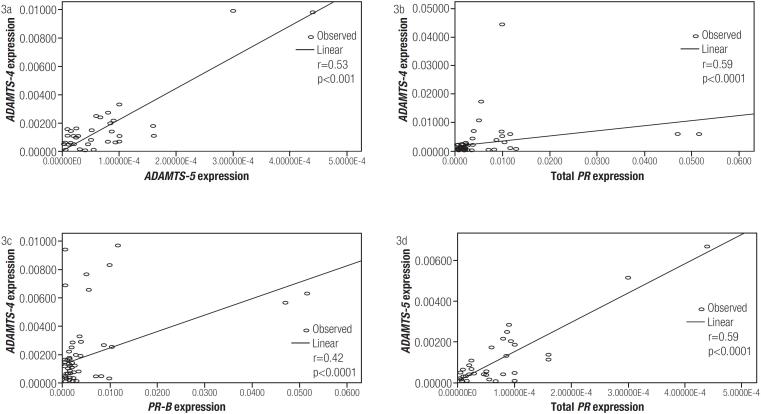
Correlations between *ADAMTS-4*, *ADAMTS-5*, and *PRs* expression in the cumulus cells.

The relationship between *ADAMTS-4* and *-5* expression levels and *PR* mRNA expression levels is shown in Figure 3. As shown in the figure, a positive relationship was observed between *ADAMTS-4* and *-5* mRNA expression levels in the study population (r = 0.53, p = 0.001). Additionally, *ADAMTS-4* expression in women with and without PCOS was remarkably correlated with total progesterone and progesterone receptor B (*PRB*) (r = 0.6, p < 0.0001, Figure 3b and r = 0.42, p < 0.0001, Figure 3c, respectively). A meaningful relationship was also observed between *ADAMTS-5* and total progesterone mRNA expression levels (r = 0.59, p < 0.0001, Figure 3d).

## DISCUSSION

ADAMTS-4 and -5 as major members of the aggrecanase group (*ADAMTS-1*, *-4*, *-5*, and *-9*) are primarily expressed from mural GCs and will be selectively localized in the ECM of expanded COCs (9,12). The results of the present study demonstrated a notable decrease in *ADAMTS-4* and *-5* expression levels in women with PCOS when compared to women with normal ovarian function, which was significantly associated with the decreased expression levels of PRs and impaired oocyte quality.

*ADAMTS-1-4*, and -*5* are functionally and structurally associated and grouped due to their overlapping activities on aggrecan, versican, and brevican degradation (13). In the current study, significant correlations observed between these proteoglycanases indicate their function in the cleavage or modification of ECM proteoglycans (14). Previous studies have revealed that ADAMTS-1 knockout mice exhibit impaired fertility because of the diminished or absent versican processing in ovarian follicles, which leads to lack of ovulation. This suggests that ADAMsTS-1 is essential for the normal folliculogenesis, ovulatory process and subsequent fertilization (15). Studies conducted during folliculogenesis process indicated an active form of ADAMTS-1 in the basement membrane surrounding growing follicles. Furthermore, versican as a common substrate for*Adamts-1, -4*, and -*5* has been identified in the follicular basement membrane from the early stage of the follicle growth. These findings suggest that the ADAMTS-1 cleavage of versican is involved in structural remodeling of the ovarian follicle and cumulus-oocyte matrix during ovulation (16).

Prior to ovulation, *ADAMTS-4, ADAMTS-5*, and versican are co-expressed within the GCs of small growing follicles and play indispensable roles at the early stages of the follicular growth and during the ovulation process (17).

Although the number of oocytes is comparable between PCOS patients and healthy women following controlled ovarian hyperstimulation during the IVF treatment, most of follicles retain their defects, which may affect the oocyte quality (3). It was previously established that the number of follicles and follicular volume were merely independent predictors of IVF/ICSI outcomes. Therefore, a healthy follicle is crucial for successful oocyte retrieval, oocyte maturation, and fertilization (18). Yung and cols. demonstrated that ADAMTS-1 expression in human CCs was significantly associated with the oocyte fertilization capacity (19). Moreover, some studies indicated that CCs gene expression predicts the oocyte quality and the subsequent pregnancy (20,21).

To our knowledge, no studies have examined the correlation between *ADAMTS-4* and *-5* expression with the oocyte quality. Here, we illustrated a significant differential expression of *ADAMTS-4* and -*5* in CCs from mature and GV oocytes in the PCOS and control groups. The results of the current study indicated a lower *ADAMTS-4* and -*5* expression in CCs from the PCOS patients compared to the controls, which was closely correlated with the reduced oocyte retrieval, oocyte maturation, and fertilization rates. Lower oocyte recovery and maturation rates, and subsequently, lower fertilization rate was observed in the PCOS patients compared to the controls. Dysregulated expression of these genes may be attributed to abnormalities in the microenvironment surrounding oocytes induced by PCOS, which alter survival and proliferation of CCs (3).

Induction of ADAMTS-1 in ovulating follicles from cows, pigs, horses, and primates has been shown to be mediated by activity of *PRs* (9,17). Moreover, *PRs* knockout mice are completely infertile because of their inability to up-regulate ADAMTS-1 and failure to ovulate even under the effect of exogenous hormones (9). Decreased expression of *PRs* was observed in GCs from the PCOS patients, which might be a sign for maturation defect or follicular arrest in GCs (6). The present study demonstrated a noticeable association between downregulated expression of *ADAMTS-4* and *-5* with *PRs* in the PCOS patients. Additionally, we previously indicated downregulation of ADAMATS-1 and -9 as two other aggrecanase family members in the PCOS patients, which was notably associated with reduced expression of *PRs* and also lower oocyte recovery and oocyte maturation rates, as well as lower fertilization rates (11).

Some previously published studies pointed out that PR mRNA expression was induced in COCs and GCs in a pattern similar to that of ADAMTS-1 and -4 (22,23). Therefore, it appears that progesterone- and PR-dependent pathways contribute to functional alternations in follicular cells during the ovulation process (24).

In summary, our study documented that diminished ADAMTS-4 and -5 expression in the PCOS patients was closely associated with the impaired oocyte quality, demonstrating that intrinsic CC dysfunction influenced the oocyte quality. Normal expression of ADAMTS-4 and -5 appears to play indispensable roles in the oocyte maturation. Dysregulation of these genes may be associated with abnormalities of endocrine and intra-ovarian paracrine factors. However, further studies are required to investigate mechanisms underlying dysregulation of these aggrecanases on ovulatory problems in PCOS.

## References

[1] Bil E, Dilbaz B, Cirik DA, Ozelci R, Ozkaya E, Dilbaz S (2016). Metabolic syndrome and metabolic risk profile according to polycystic ovary syndrome phenotype. J Obstet Gynaecol Res.

[2] Ibáñez L, Oberfield SE, Witchel S, Auchus RJ, Chang RJ, Codner E (2017). An International Consortium Update: pathophysiology, diagnosis, and treatment of polycystic ovarian syndrome in adolescence. Horm Res Paediatr.

[3] Xiao S, Li Y, Li T, Chen M, Xu Y, Wen Y (2014). Evidence for decreased expression of ADAMTS-1 associated with impaired oocyte quality in PCOS patients. J Clin Endocrinol Metab.

[4] Demircan K, Cömertoğlu İ, Akyol S, Yiğitoğlu BN, Sarıkaya E (2014). A new biological marker candidate in female reproductive system diseases: Matrix metalloproteinase with thrombospondin motifs (ADAMTS). J Turk Ger Gynecol Assoc.

[5] Mattison DR (2015). Computational Methods for Reproductive and Developmental Toxicology.

[6] Artimani T, Saidijam M, Aflatoonian R, Amiri I, Ashrafi M, Shabab N (2015). Estrogen and progesterone receptor subtype expression in granulosa cells from women with polycystic ovary syndrome. Gynecol Endocrinol.

[7] Demircan K (2012). A Multi-Functional Gene Family From Arthritis to Cancer&58; A Disintegrin-Like Metalloproteinase with Thrombospondin Type-1 Motif (ADAMTS). Journal of Clinical and Analytical Medicine.

[8] Russell DL, Doyle KM, Ochsner SA, Richards JS, Sandy JD, Mittaz-Crettol L (2003). Localization of ADAMTS-1 and proteolytic cleavage of versican during cumulus matrix expansion and ovulation. Reprod Fertil Dev.

[9] Russell DL, Brown HM, Dunning KR (2015). ADAMTS proteases in fertility. Matrix Biol.

[10] Karakose M, Demircan K, Tutal E, Demirci T, Arslan MS, Sahin M (2016). Clinical significance of ADAMTS1, ADAMTS5, ADAMTS9 aggrecanases and IL-17A, IL-23, IL-33 cytokines in polycystic ovary syndrome. J Endocrinol Invest.

[11] GohariTaban S, Amiri I, Soleimani Asl S, Saidijam M, Yavangi M, Khanlarzadeh E (2019). Abnormal expressions of ADAMTS-1, ADAMTS-9 and progesterone receptors are associated with lower oocyte maturation in women with polycystic ovary syndrome. Arch Gynecol Obstet.

[12] Wen J, Zhu H, Leung PC (2013). Gonadal steroids regulate the expression of aggrecanases in human endometrial stromal cells in vitro. J Cell Mol Med.

[13] Demircan K, Topcu V, Takigawa T, Akyol S, Yonezawa T, Ozturk G (2014). ADAMTS4 and ADAMTS5 knockout mice are protected from versican but not aggrecan or brevican proteolysis during spinal cord injury. Biomed Res Int.

[14] Kelwick R, Desanlis I, Wheeler GN, Edwards DR (2015). The ADAMTS (A Disintegrin and Metalloproteinase with Thrombospondin motifs) family. Genome Biol.

[15] Brown HM, Dunning KR, Robker RL, Pritchard M, Russell DL (2006). Requirement for ADAMTS-1 in extracellular matrix remodeling during ovarian folliculogenesis and lymphangiogenesis. Develop Biol.

[16] Brown HM, Dunning KR, Robker RL, Boerboom D, Pritchard M, Lane M (2010). ADAMTS1 cleavage of versican mediates essential structural remodeling of the ovarian follicle and cumulus-oocyte matrix during ovulation in mice. Biol Reprod.

[17] Richards JS, Hernandez-Gonzalez I, Gonzalez-Robayna I, Teuling E, Lo Y, Boerboom D (2005). Regulated expression of ADAMTS family members in follicles and cumulus oocyte complexes: evidence for specific and redundant patterns during ovulation. Biol Reprod.

[18] Merce LT, Bau S, Barco MJ, Troyano J, Gay R, Sotos F (2006). Assessment of the ovarian volume, number and volume of follicles and ovarian vascularity by three-dimensional ultrasonography and power Doppler angiography on the HCG day to predict the outcome in IVF/ICSI cycles. Hum Reprod.

[19] Yung Y, Maman E, Konopnicki S, Cohen B, Brengauz M, Lojkin I (2010). ADAMTS-1: a new human ovulatory gene and a cumulus marker for fertilization capacity. Mol Cell Endocrinol.

[20] Adriaenssens T, Wathlet S, Segers I, Verheyen G, De Vos A, Van der Elst J (2010). Cumulus cell gene expression is associated with oocyte developmental quality and influenced by patient and treatment characteristics. Hum Reprod.

[21] Wathlet S, Adriaenssens T, Segers I, Verheyen G, Van de Velde H, Coucke W (2011). Cumulus cell gene expression predicts better cleavage-stage embryo or blastocyst development and pregnancy for ICSI patients. Hum Reprod.

[22] Richards JS (2005). Ovulation: new factors that prepare the oocyte for fertilization. Mol Cell Endocrinol.

[23] Russell DL, Doyle KM, Ochsner SA, Sandy JD, Richards JS (2003). Processing and localization of ADAMTS-1 and proteolytic cleavage of versican during cumulus matrix expansion and ovulation. J Biol Chem.

[24] Sriraman V, Sinha M, Richards JS (2010). Progesterone receptor-induced gene expression in primary mouse granulosa cell cultures. Biol Reprod.

